# Comparison and bias analysis of medically attended acute gastroenteritis incidence estimates derived from electronic health record surveillance versus cross-sectional surveys

**DOI:** 10.1371/journal.pone.0323425

**Published:** 2025-05-19

**Authors:** Laura E. Calderwood, Rachel M. Burke, Claire P. Mattison, Mark A. Schmidt, Holly C. Groom, Judy Donald, Aron J. Hall, Sara A. Mirza

**Affiliations:** 1 Division of Viral Diseases, Centers for Disease Control and Prevention, Atlanta, GeorgiaUnited States of America; 2 Cherokee Nation Operational Solutions, Tulsa, Oklahoma, United States of America; 3 Center for Health Research, Kaiser Permanente Northwest, Portland, Oregon, United States of America; 4 Kaiser Permanente Bernard J Tyson School of Medicine, Pasadena, California, United States of America; United States Environmental Protection Agency, UNITED STATES OF AMERICA

## Abstract

Disease burden studies commonly use data from electronic health records (EHRs) or community surveys. Quantitative bias assessments of these study designs are needed. We compared two studies on acute gastroenteritis (AGE) burden conducted in an integrated healthcare system in Oregon and Washington, USA. EHRs were used to identify AGE patients who sought care during July 2014 – June 2016 and determine the incidence of medically attended AGE (MAAGE). Members from the same health care system were surveyed during September 2016 – September 2017 to estimate community AGE incidence. MAAGE incidence was calculated using the rate of reported healthcare seeking among survey respondents and compared to the estimate derived from the EHR study. Survey respondents’ EHR data were used to conduct a bias analysis. MAAGE incidence from survey respondents was 6.1 times higher than the EHR derived MAAGE estimate. Among survey respondents who self-reported contacting KPNW for an AGE episode, 36.3% had an AGE-coded encounter in the EHR during the same timeframe, and among those who reported no contact (either no AGE or AGE without medical attention), 2.6% did have an AGE-coded encounter. Potential noninfectious explanations for symptoms were reported by 35% of ill survey respondents. We quantify misclassification bias in both studies and discuss other potential sources of bias. Researchers should consider these biases when designing disease burden studies and consider including sensitivity analyses in published work.

## Introduction

Acute gastroenteritis (AGE) is a major cause of illness globally, with an estimated 179 million AGE illnesses occurring annually in the United States (US) alone [1]. Although most AGE does not require medical attention (in the US, ~ 20% of AGE cases report seeking medical care), the high incidence of disease results in a substantial burden on the health care system [2,3]. Accurate estimates of AGE incidence, including medically attended and self-managed cases, are important to understand the burden of illness and assess the potential impacts of public health interventions, such as vaccines.

To estimate AGE burden, some studies focus on the healthcare system through active surveillance or analysis of electronic health records (EHRs). EHR studies are highly cost efficient and have the benefit of capturing a large population; however, their accuracy is contingent on the coding practices of healthcare workers [4]. Other studies measure AGE in the community to capture the full spectrum of disease including self-managed cases, through cross sectional surveys or longitudinal cohort surveillance. Compared to EHR studies, community studies require more resources and may present difficulties in achieving statistical power, as detection of AGE cases is dependent on the underlying prevalence. Among community studies, cross-sectional surveys are less resource intensive, but may be prone to nonresponse bias and recall bias [5,6]. Longitudinal cohorts produce the richest data; however, these studies are the most resource intensive, and participation may influence behavior among cohort members [7,8].

It is important to assess the potential biases affecting different study designs to better contextualize and interpret epidemiological estimates. Two studies of AGE incidence were conducted in an integrated healthcare system in the Pacific Northwest region of the US. One study used prospective EHR surveillance, while the second surveyed community members enrolled in the same healthcare system using random weekly samples. We present a comparison of estimates derived from each study, and an analysis of various biases that may contribute to discrepancies between the two.

## Materials and methods

This analysis compares estimates of AGE incidence from two previously published studies among Kaiser Permanente Northwest (KPNW) members [9–11]. KPNW is an integrated healthcare system that serves over 600,000 members, representing approximately 24% of the underlying population in northwestern Oregon and southwestern Washington. Overall, the demographic makeup of KPNW members is similar to that of the region, with notable differences including a slightly older age distribution and lower proportion of Medicaid recipients among members [10,12].

In brief, the Medically Attended Acute Gastroenteritis (MAAGE) study identified cases of medically attended AGE using active EHR surveillance during April 1, 2014 – September 30, 2016. Case records were identified using codes from the International Classification of Diseases (ICD) by flagging selected ICD-9 (April 2014 – September 2015) and ICD-10 codes (April 2014 – September 2016) in the EHR. Selected ICD-9 and 10 codes included cause-unspecified AGE, as well as specified viral, bacterial, and parasitic infections [10]. EHR data from remote visits (telephone or email), outpatient visits, emergency department visits, and hospital admissions were included. The deidentified dataset was accessed December 17, 2019, and restricted to July 1, 2014 – June 30, 2016 to control for seasonal variation. AGE-associated medical encounters occurring within 30 days of each other were condensed into a single episode for analysis [9].

The Community Acute Gastroenteritis (CAGE) study surveyed KPNW members to generate 30-day prevalence and annualized incidence estimates of AGE and describe the proportion of AGE cases seeking healthcare in the KPNW member population. Age-stratified, random, weekly samples of KPNW members were selected during October 1, 2016 – September 30, 2017. KPNW members who were in hospice care, non-English speaking, cognitively impaired, or who had previously declined participation in KPNW research were ineligible for selection. All selected members were contacted by postcard and email, and a subset received phone calls; those who provided informed consent and completed the online survey received a $10 gift card (an additional $10 was provided to individuals who provided a stool specimen for an associated sub-study). The age group used for sampling was the only data available for non-participants. The survey included demographic questions and information on AGE symptoms within the previous 30 days. Participants with AGE were asked about frequency and duration of vomiting and diarrhea, chronic enteric illness, non-infectious potential causes of their symptoms, and whether or not the contacted KPNW for their illness. AGE was defined as any vomiting (>1 episode within 24 hours) or diarrhea (>3 loose stools in any 24-hour period), not related to a chronic enteric illness. All calculations were weighted on age and other demographic factors available to KPNW to account for differences in nonresponse rates across groups. Community incidence was calculated by multiplying 30-day prevalence by 365/30 to yield the average number of AGE illnesses per person-year [11].

### Analysis

To compare the results of the MAAGE and CAGE studies, we generated age-stratified estimates of medically attended AGE incidence for CAGE survey respondents and compared these estimates to the previously published estimates from MAAGE. Within age strata, medically attended AGE incidence was calculated for the CAGE study using the number of CAGE survey respondents who reported contacting KPNW for AGE illness (remote or in-person visits) in the previous 30 days. We calculated the number of episodes per year for each age strata based on KPNW membership to adjust for age-stratification within the CAGE sampling methodology. Overall medically attended AGE incidence was calculated using the estimated total episodes. For simplicity, community incidence estimates were only weighted for age in this analysis. Among CAGE survey respondents, we assessed the agreement of survey responses and AGE ICD-10 codes by extracting EHR data from KPNW medical encounters of any kind during the study period, and classified respondents as (a) having reported contacting KPNW for an AGE illness in the previous 30 days or not and (b) having an AGE encounter detected in the EHR in the previous 30 days or not. Although both study methods may be subject to misclassification, we quantified agreement by estimating the sensitivity and specificity of the AGE ICD-10 codes used to enroll participants in the MAAGE study against the survey responses. We then created a bias-adjusted incidence estimate for the MAAGE study by reclassifying medically attended AGE cases and non-cases in the base population using the formula $A=\;\frac{SpA^{*}-(1-Sp)B^{*}}{SeSp-(1-Se)(1-Sp)}$ where A is the number of true cases, A* is the number of classified cases, B* is the KPNW membership minus the classified cases, Sp is the specificity of ICD-10 codes, and Se is the sensitivity of ICD-10 codes [13].

We conducted sensitivity analyses of both medically attended and community AGE incidence estimates from the CAGE study by reclassifying cases based on the following:

- ICD-10 codes for vomiting not included in the MAAGE study protocol (R11.2 and R11.10)
- alternate explanations for symptoms, i.e., pregnancy, alcohol use, medications, food intolerances, or other ongoing digestive issues
- a stricter definition of AGE in which respondents with a single episode of vomiting and no loose stools were treated as non-cases.

Analyses were conducted in SAS (Cary, NC, v 9.4), Excel (Microsoft Inc.), and the R Environment for Statistical Computing (version 3.4.2).

### Ethics statement

This project was reviewed and approved by the KPNW Institutional Review Board (FWA00002344). The institutional review board granted a waiver of informed consent for the data collected from the EHR as part of the MAAGE study, and individuals who have opted out of Kaiser Permanente Research activities were excluded from the dataset. All participants provided informed consent to participate in the CAGE study; for children under 18 years of age, legal guardians provided consent and completed the survey on their child’s behalf.

## Results

### Comparison of incidence estimates from MAAGE and CAGE studies

During July 2014 – June 2016, 42,030 medically attended AGE episodes were identified by EHR in the MAAGE study, corresponding to an incidence of 4.1 medically attended AGE cases per 100 person-years (PY) [9].

Of 28,080 KPNW members selected for the CAGE study during September 2016 – September 2017, 3,894 (13.8%) completed the survey (Table 1). Participation was highest among individuals aged ≥65 years (17.8%) and lowest among adults ages 18–64 (12.5%). Excluding 62 survey respondents with chronic enteric illness and no vomiting, 395 (10.4%) reported illness involving vomiting or diarrhea in the previous 30 days. The estimated incidence of community AGE among survey respondents was 127 cases per 100 PY [11]. Eighty-two (26.2%) AGE cases reported contacting KPNW, corresponding to an incidence of 24.7 medically attended AGE cases per 100 PY.

**Table 1:** Response rates, acute gastroenteritis (AGE) prevalence, and healthcare seeking among Kaiser Permanente members selected for the CAGE survey, 2016-2017.

Age group	Total sampled	Total respondents	Reported AGE symptoms^a^	AGE incidence^b^	Contacted KPNW
< 2 years	3,224^c^	197	19 (9.6%)	117.5	7 (36.8%)
2 - 4 years		276	46 (16.7%)	200.7	13 (28.3%)
5 - 17 years	2,184	334	36 (10.8%)	132.1	10 (27.8%)
18 - 64 years	17,888	2,234	239 (10.7%)	130.2	40 (16.7%)
65 + years	4,784	853	55 (6.4%)	78.5	12 (21.8%)
Overall	28,080	3,894	395	123.7	82 (26.2%)

^a^Excluding individuals with chronic enteric illness

^b^Incidence per 100 person-years

^c^The age breakdown of children <5 sampled in the CAGE study was not available for analysis

We found a six-fold difference in our estimates of medically attended AGE incidence (4.1 vs. 24.7 cases per 100 PY) derived from the two studies. Age-stratified medically attended AGE incidence estimates from both studies are shown in Table 2 and S1 Fig. The discrepancy between estimates was highest in children 5–17 years, for whom CAGE survey incidence estimates were 14 times higher than EHR-derived estimates from the MAAGE study (Table 2). Adults aged 65 + and children <2 years had the smallest discrepancies, although survey-based estimates were still ~3 times higher than those from the EHR study in both age groups.

**Table 2:** Comparison of estimated incidence of medically attended acute gastroenteritis per 100PY derived from EHR surveillance (MAAGE study; 2014-2016) and a community survey (CAGE study; 2016-2017) among members of Kaiser Permanente Northwest, by age group.

Age Group	Incidence From MAAGE	Incidence From CAGE	Ratio of incidence estimates
< 2 years	13.2	43.3	3.3
2–4 years	5.8	56.7	9.8
5–17 years	2.6	36.7	14.0
18–64 years	3.5	21.8	6.2
65 + years	6.1	17.1	2.8
All ages	**4.1**	**24.7**	**6.1**

### Analysis of EHR sensitivity and specificity in the CAGE study

The medical records of3,714 (95%) CAGE survey respondents were eligible for analysis. Of the 80 individuals who self-reported contacting KPNW for an AGE episode, 29 had an AGE-coded encounter in the EHR during the same timeframe (sensitivity = 36.3%). Although not statistically significant, sensitivity of ICD-10 codes was higher among those who reported in-person visits (24/58; 41.4%) compared to those who reported remote visits (5/17; 29.4%). Of the 3,634 individuals who reported no contact with KPNW, 96 had an AGE-coded encounter during this timeframe, including 81 who reported no AGE and 15 who reported AGE without medical attention (specificity = 97.4%). ICD-10 codes had a positive predictive value of 23.2% and a negative predictive value of 98.6%. Our numbers were too small to examine study agreement by age or other demographic characteristics.

To better understand the low detection of self-reported AGE encounters using ICD-10 codes, we examined the ICD-10 codes of non-AGE-coded KPNW encounters among respondents who reported contacting KPNW but did not have a corresponding AGE-coded encounter. Of 51 such individuals, 45 (88%) had KPNW encounters that were not flagged as AGE. Among these, the 15 most common ICD-10 codes, listed in S1 Table, included a mix of enteric and non-enteric symptoms and diagnoses. Potentially AGE-related ICD-10 codes that were not flagged in our MAAGE study protocol included generalized and unspecified abdominal pain, unspecified vomiting, and hemorrhage and other diseases of the anus and rectum. Non-AGE codes that may have been accompanied by vomiting or loose stool included anxiety disorder, upper respiratory infection, and gastro-esophageal reflux disease. Reclassifying encounters with unspecified vomiting as AGE encounters resulted in an additional 7 survey respondents with AGE encounters; as all 7 had reported contacting KPNW for AGE, sensitivity increased to 45%, and specificity was unchanged.

### Bias adjustment of MAAGE incidence estimates

Applying the sensitivity (36.3%) and specificity (97.4%) estimates from survey respondents to the MAAGE study provided a bias-adjusted estimate of 44,883 medically attended AGE cases in the KPNW population over the two-year period, and an incidence of 4.3 medically attended AGE episodes per 100PY. This bias-adjusted incidence estimate was 7% higher than the original MAAGE study estimate of 4.1 episodes per 100PY. Bias-adjusted MAAGE incidence estimates for a range of sensitivities and specificities are shown in Table 3.

**Table 3:** Bias-corrected estimates of medically attended acute gastroenteritis (AGE) incidence (per 100 person-years) in the MAAGE study, using a range of possible values for the sensitivity and specificity of ICD-9 and ICD-10 codes for detecting AGE encounters.

		Sensitivity				
		26.3%	31.3%	36.3%^a^	41.3%	46.3%
Specificity	96.4%^b^	2.04	1.67	1.42	1.23	1.08
	**97.4%** ^a^	6.17	5.10	4.34	3.78	3.35
	98.4%	9.97	8.29	7.10	6.20	5.51
	99.4%	13.47	11.28	9.70	8.51	7.58

^a^Sensitivity (36.3%) and specificity (97.4%) were calculated using CAGE survey responses for whether AGE cases contacted Kaiser Permanente as the ‘gold standard’ compared to health records.

^b^Bias corrections for specificity values below 96% resulted in invalid (negative) incidence estimates

### Sensitivity analysis of CAGE incidence estimates

Survey respondents with AGE were reclassified based on symptom status and attribution of symptoms to other causes. Nineteen cases reported only a single vomiting episode and no loose stools; reclassification of these participants as non-cases decreased the incidence to 118 community AGE episodes and 23.3 medically attended AGE episodes per 100PY.

Respondents with vomiting or diarrhea were asked if they attributed their symptoms to certain other causes (Table 4). Among respondents who said they had not been diagnosed with a chronic enteric illness, 30 (8%) AGE cases believed their illness may be due to an ongoing illness such as irritable bowel disease or colitis. Another 108 AGE cases (27%) attributed their symptoms to other alternate causes. Reclassifying these 138 cases as non-cases decreased incidence to 80.3 community AGE episodes and 15.4 medically attended AGE episodes per 100PY.

**Table 4:** Attribution of vomiting and diarrhea to noninfectious and non-acute causes by CAGE survey respondents who met the case definition of acute gastroenteritis, 2016–2017.

Question: Do you think the loose stools or vomiting you experienced were due to any of the following? (Select all that apply)	Yes n(%)
A long-term illness such as irritable bowel syndrome or colitis	30 (8%)
A food allergy or intolerance	68 (17%)
Medication or treatment	33 (9%)
Pregnancy	3 (1%)
Alcohol consumption	9 (2%)
Other ongoing digestive issues	59 (15%)
Any of the above	138 (35%)

## Discussion

The estimated incidence of medically attended AGE was 6 times higher when extrapolating from the CAGE cross-sectional surveys compared to the estimate derived from medical records in the MAAGE study. Although the CAGE study was primarily designed to estimate community AGE prevalence and not medically attended AGE incidence, and some year-to-year variation in AGE incidence is typical, we expected the two estimates to be closer and conducted a bias analysis to determine possible explanations for the discrepancy. The specificity of ICD-10 codes used in the MAAGE study among CAGE survey respondents was high at 97% and sensitivity was low, at just 36%. Adding ICD-10 codes for vomiting improved sensitivity without decreasing specificity. However, our bias-adjusted medically attended AGE incidence estimate of 4.3 per 100PY was similar to the unadjusted estimate and did not account for the discrepancy between study designs. We then reclassified CAGE survey respondents based on stricter AGE definitions. Reclassifying AGE cases who reported only a single vomiting episode as non-cases reduced the incidence estimates by about 5%, and reclassifying those who reported a potential non-infectious explanation for their AGE symptoms decreased our incidence estimates by 38%. However, even with these corrections, our adjusted CAGE-derived estimate for medically attended AGE incidence is still 3.8 times higher than the MAAGE study estimate. The potential impact of biases associated with these two study designs is summarized in Table 5.

**Table 5:** Summary of potential sources of bias in the MAAGE and CAGE studies and the likely direction of bias resulting from each source.

Scenario	Description of potential bias
**Misclassification**	
Medically attended AGE encounters are not flagged as AGE in EHR	Medically attended AGE incidence estimate^a^ is biased DOWN
AGE is reported on survey when the nature of vomiting or diarrhea does not constitute an AGE episode	Community AGE incidence estimate^b^ is biased UP
Legal guardians responding to the survey on behalf of older children may be unaware of mild AGE symptoms	Community AGE incidence estimate^b^ is biased DOWN; proportion of community AGE cases that seek healthcare^c^ is biased UP
Survey respondent experienced recent AGE, but misremembered whether it was in the previous 30 days	Community AGE incidence estimate^b^ could be biased UP OR DOWN
Respondent experienced AGE, but AGE was not the primary reason for medical encounter. If AGE was mentioned, the patient and provider reports may not align	Medically attended AGE incidence estimate^a^ and proportion of community AGE cases that seek healthcare^c^ could be biased UP OR DOWN
**Selection (nonresponse) bias**	
Individuals more likely to complete a survey about AGE if recently ill with AGE	Community AGE incidence estimate^b^ is biased UP
Individuals who have high contact with KPNW more likely to complete a survey from them AND more likely to seek care for AGE	Proportion of community AGE cases that seek healthcare^c^ is biased UP

AGE = acute gastroenteritis

^a^Medically attended AGE incidence estimate from the MAAGE study

^b^Community AGE incidence estimate from the CAGE study

^c^Care seeking was measured in the CAGE survey as AGE cases who reported contacting Kaiser Permanente for their illness

### Bias in EHR studies

Generally, EHR studies are subject to bias in the form of misclassification; this bias may result in either over- or underestimates. Employing an incomplete list of ICD-10 codes that indicate the disease of interest will result in low sensitivity and may result in an underestimation of the burden of disease on the healthcare system. Conversely, using ICD-10 codes that are non-specific to the diagnosis under study may bias burden estimates upwards. Although adding ICD-10 codes for vomiting did not decrease specificity in our sample, it is possible that improving sensitivity of an ICD-10 code list may decrease specificity. Despite the low sensitivity (36%) and seemingly high specificity (97%) in our comparison of CAGE participants’ survey responses to their medical records, we calculated a similar bias-adjusted incidence estimate for the MAAGE study due to the large population at risk leading to a high potential for false positives which largely offsets the potential for false negatives. However, in the absence of a reliable gold-standard, our estimates are meant to be a demonstration of poor study agreement rather than a true measure of sensitivity and specificity and, as shown in Table 3, incremental differences in sensitivity and specificity could lead to large variations in the direction of bias. Of note, it is unlikely that an EHR entry would exist for a medical encounter that never occurred; therefore, we attribute the false positives in our study to individuals who did have medical encounters for other reasons, with incorrect coding. There may also be instances in which the false positives described here are attributable to differences in our definition of AGE, and are in fact valid AGE medical encounters; for example, an illness causing abdominal cramps and nausea would not be captured as an AGE episode in the CAGE study, but could lead to an AGE-coded medical encounter.

While this analysis did not explain the discrepancy between estimates from the two study designs, it is important for those designing EHR studies to consider the outsized impact of specificity on the accuracy of results. Both low sensitivity and specificity of EHR codes have also been found in studies of kidney and heart disease [14,15]. Horsky et al. tested clinicians with standardized scenarios and found clinicians often entered inappropriate codes or omitted relevant codes, and the search terms used to query the EHR were variable [4]. As EHR studies are used to answer a wide array of questions about healthcare (e.g., disease burden, care practices, and risk factor analyses) due to their statistical power and cost-effectiveness, researchers should be conscientious of the potential impact of misclassification in all EHR studies. Those interpreting EHR studies should also consider that results may not be widely generalizable, as those using a particular healthcare system may not represent the general population.

### Bias in community surveys

Surveys and cross-sectional studies are subject to both misclassification and non-participation bias. In our survey, misclassification may have taken a few forms. First, respondents may have met the AGE case definition despite the cause of their symptoms being outside the scope of research interest, causing an overestimation of community incidence. We asked if ill survey respondents believed their symptoms of vomiting or diarrhea could be attributed to pregnancy, alcohol use, chronic enteric illness, medications, food intolerances, or other ongoing digestive issues, but initially only excluded those with chronic enteric illnesses from analysis. When excluding those who reported any alternate explanations for their symptoms, our community and medically attended AGE incidence estimates were reduced by 38%. Although these cases do meet the AGE case definition, they may explain a portion of the discrepancy in incidence estimates from the two studies since their symptoms may be unlikely to be recorded as AGE in the EHR when seeking medical attention, even with our inclusion of broad ICD codes such as “Diarrhea” and “Other and unspecified noninfectious gastroenteritis and colitis”. By examining the ICD-10 codes of ill survey respondents with non-AGE coded encounters, we also found a few additional potential causes of vomiting or diarrhea, such as anxiety and acute respiratory infection. Of note, misclassification could cause underestimation if respondents incorrectly assume their experience is not what the researcher is seeking. Second, our study asked specific questions about an event that may have occurred up to 30 days before survey completion and was therefore subject to recall bias. Participants may have forgotten the details of their illness leading to potential bias in either direction. For example, if a respondent remembers an acute enteric illness as occurring more recently than it did in truth (i.e., they report an episode within the last 30 days when it occurred more than 30 days ago), our prevalence and corresponding incidence estimates would be biased upwards. We did not have data available to analyze the effects of recall bias in this study. Third, we asked legal guardians to complete the survey on behalf of all participants under 18 years of age, which may have introduced additional misclassification of disease status, particularly among 5–17 year olds – the group with the highest discrepancy between EHR and survey derived estimates. While legal guardians are likely to be able to accurately report whether their child had a medical visit for AGE, they may be unaware if their child experienced a mild illness, biasing the proportion of ill 5-17 year olds who sought care upwards. This type of misclassification would not change our survey-derived MAAGE incidence estimate, as the increased proportion seeking care is mathematically cancelled out by a lower community AGE incidence; it may, however, result in inaccuracies in our other reported results.

None of these sensitivity analyses and adjustments fully explained the difference in estimates between the MAAGE and CAGE studies. With an overall response rate of 13%, it is likely the remaining difference in estimates is due to participation bias caused by differential nonresponse rates based on recent illness or care-seeking behaviors among KPNW members selected for CAGE study recruitment. Because the communications used for recruitment referenced gastroenteric illness, those who were recently ill with AGE may have been more likely to respond to the survey, inflating our estimate of community AGE incidence. Selection bias, in this case due to nonresponse, is a separate issue from misclassification [16–18], and is likely among the largest contributors to bias in our measures of disease burden. In the published results from the CAGE study, responses were weighted for demographic factors to account for differences in nonresponse rates, and the weighted incidence estimate (127 per 100PY) was similar to the unweighted estimate (124 per 100PY) [11]. While it is important to account for these demographic differences, it does not address nonresponse bias that occurs if participation rates are directly associated with the outcome of interest. The magnitude of such bias can be great, and due to the lack of information on the health status of nonparticipants, it is difficult to quantify. However, our 30-day AGE prevalence of 10.4% (95%CI: 9.3–11.6%) is somewhat higher than the prevalence estimated from the National Health and Nutrition Examination Survey (NHANES) of 8.3% (95%CI: 7.8–8.8%) [19]. As a survey with a broader scope administered to an unaffiliated population, NHANES is unlikely to be highly susceptible to nonresponse bias based on AGE outcome.

Nonresponse bias may have also affected our estimate of the proportion of AGE cases who seek care. Individuals with frequent healthcare contact may feel more connected to KPNW and be more inclined to participate in research activities. Therefore, the proportion of care-seeking among AGE cases may be higher among survey respondents than in the general population, which would inflate our survey-derived MAAGE incidence estimate. The percentage of AGE cases who sought care among our survey respondents (18%) was similar to that found in other US studies (19–20%) [2,3]; however, these studies followed a similar community survey design and may have been subject to similar biases. With the increasing difficulty of getting randomly selected individuals to engage in research activities, researchers should be highly aware of the potential impacts of nonresponse bias in their research strategy.

This analysis demonstrates the potential impact of biases to which EHR studies and surveys are susceptible. However, this analysis is subject to a few limitations. Firstly, while we were able to compare data from the same source population, these data came from different time frames; year-to-year variation in circulating AGE pathogens may explain some differences between the two studies. Secondly, we did not have data available to quantitatively assess every possible bias that may have affected our estimates, such as nonresponse bias or recall bias; as the effects of misclassification quantified in this analysis were modest compared to the overall discrepancy between study designs, nonresponse bias and recall bias among survey respondents likely have a large impact on overall disease burden estimates. Differences in the definition of AGE as well as the exclusion of AGE cases with chronic enteric illnesses may also contribute to discrepancies between study designs, as there may be differences in the rates of self-reported illness compared to those detected in medical records. The design of the CAGE survey, in capturing prevalent cases which may have begun before the 30-day recall window, may have also contributed to the inflated incidence estimate from the CAGE study. This study was not powered to assess differences in sensitivity and specificity of ICD-10 codes among survey respondents by age, level of care, or other factors that may affect the way clinicians complete records. As care-seeking behavior patterns shift and telehealth options become more popular, further research is needed to determine how the level of care may affect the susceptibility of EHR studies to bias. Additionally, we used survey responses as the “gold standard” to calculate sensitivity and specificity of our MAAGE ICD-10 code protocol as we believed ill individuals would be likely to correctly recall whether they sought care from KPNW for their illness; however, we acknowledge that while this comparison provides insight into the level of agreement between the two study designs, survey responses are also affected by misclassification bias and contain imperfect data. While both EHR studies and community surveys are relatively common in the literature, our study presents a rare opportunity to compare the two within the same population.

## Conclusions

Studies that collect information through community surveys and those that leverage electronic medical records for disease surveillance contribute valuable knowledge on disease burden and epidemiologic trends. In comparing results from two studies in the same population (members of a specific healthcare system), we found substantial discrepancies and identified multiple potential sources of bias. Researchers should consider the impacts of misclassification and nonparticipation bias when employing these study designs and consider including sensitivity analyses in published work.

## Supporting information

S1 Table Top ICD-10 codes for non-AGE coded medical encounters from CAGE survey respondents who reported contacting Kaiser Permanente for vomiting or diarrhea, 2016–2017. (DOCX)

S1 Fig Estimated incidence of medically attended acute gastroenteritis per 100PY derived from electronic health record (EHR) surveillance (MAAGE study; 2014–2016) and a community survey (CAGE study; 2016–2017) among members of Kaiser Permanente Northwest, by age group. The numeric call outs indicate the ratio of survey-derived incidence estimates to EHR incidence estimates.
(DOCX)
