# Supplementary material for: Comparison and bias analysis of medically attended acute gastroenteritis incidence estimates derived from electronic health record surveillance versus cross-sectional surveys
Source: PLoS One. 2025 May 19;20(5):e0323425. doi: 10.1371/journal.pone.0323425 (PMC12087988; doi:10.1371/journal.pone.0323425)
Supplement: S1 Table — (DOCX) [file pone.0323425.s001.docx]

**S1 Table. Top ICD-10 codes for non-AGE coded medical encounters from CAGE survey respondents who reported contacting Kaiser Permanente for vomiting or diarrhea, 2016-2017.**

| ICD-10 code | Diagnosis | Frequency |
| --- | --- | --- |
| Z79.01 | Long term (current) use of anticoagulants | 17 |
| I48.91 | Unspecified atrial fibrillation | 11 |
| R50.9 | Fever, unspecified | 9 |
| R10.84 | Generalized abdominal pain | 8 |
| N17.9 | Acute kidney failure, unspecified | 7 |
| R10.9 | Unspecified abdominal pain | 7 |
| Z71.89 | Other specified counseling | 7 |
| N18.6 | ESRD | 6 |
| R05 | Cough | 6 |
| R11.10 | Vomiting, unspecified | 6 |
| Z99.2 | Dependence on renal dialysis | 6 |
| F33.1 | Major depressive disorder, recurrent, moderate | 5 |
| F41.9 | Anxiety disorder, unspecified | 5 |
| J06.9 | Acute upper respiratory infection, unspecified | 5 |
| K21.9 | Gastro-esophageal reflux disease without esophagitis | 5 |
| K62.5 | Hemorrhage of anus and rectum | 5 |
| K62.89 | Other specified diseases of anus and rectum | 5 |
