# Supplementary material for: Comparison and bias analysis of medically attended acute gastroenteritis incidence estimates derived from electronic health record surveillance versus cross-sectional surveys
Source: PLoS One. 2025 May 19;20(5):e0323425. doi: 10.1371/journal.pone.0323425 (PMC12087988; doi:10.1371/journal.pone.0323425)
Supplement: S1 Fig — The numeric call outs indicate the ratio of survey-derived incidence estimates to EHR incidence estimates. (DOCX) [file pone.0323425.s002.docx]

**S1 Fig. Estimated incidence of medically attended acute gastroenteritis per 100PY derived from electronic health record (EHR) surveillance (MAAGE study; 2014-2016) and a community survey (CAGE study; 2016-2017) among members of Kaiser Permanente Northwest, by age group.** The numeric call outs indicate the ratio of survey-derived incidence estimates to EHR incidence estimates.
